# Metataxonomic Characterization of Enriched Consortia Derived from Oil Spill-Contaminated Sites in Guimaras, Philippines, Reveals Major Role of *Klebsiella* sp. in Hydrocarbon Degradation

**DOI:** 10.1155/2023/3247448

**Published:** 2023-09-25

**Authors:** Kiara Nicole D. Rodriguez, Russel T. Santos, Michael Joseph M. Nagpala, Rina B. Opulencia

**Affiliations:** ^1^Department of Biology, University of the Philippines Manila, Ermita, Manila 1000, Philippines; ^2^Genetics and Molecular Biology Division, Institute of Biological Sciences, University of the Philippines Los Baños, College, Laguna 4031, Los Baños, Philippines; ^3^Microbiology Division, Institute of Biological Sciences, University of the Philippines Los Baños, College, Laguna 4031, Los Baños, Philippines

## Abstract

Oil spills are major anthropogenic disasters that cause serious harm to marine environments. In the Philippines, traditional methods of rehabilitating oil-polluted areas were proven to be less efficient and cause further damage to the environment. Microbial degradation has poised itself to be a promising alternative to those traditional methods in remediating oil spills. Hence, the present study aimed to enrich and characterize hydrocarbon-degrading microbial consortia from oil-contaminated regions in Guimaras Island for potential use in bioremediation. A total of 75 soil samples were obtained and used as inoculum for the enrichment for hydrocarbon degraders. Afterwards, 32 consortia were recovered and subjected to the 2,6-DCPIP assay for biodegradation ability on four types of hydrocarbons: diesel, xylene, hexane, and hexadecane. The consortia that obtained the highest percent degradation for each of the four hydrocarbons were “B2” (92.34% diesel degraded), “A5” (85.55% hexadecane degraded), “B1” (74.33% hexane degraded), and “B7” (63.38% xylene degraded). Illumina MiSeq 16S rRNA gene amplicon sequencing revealed that the dominant phyla in all consortia are Pseudomonadota (previously Proteobacteria), followed by Bacillota (previously Firmicutes). Overall, the amplicon sequence variants (ASVs) retrieved were mainly from the *Gammaproteobacteria* class, in which many hydrocarbon-degrading bacteria are found. Predictive functional profiling of the consortium showed the presence of genes involved in the degradation of recalcitrant hydrocarbon pollutants. Fatty acid metabolism, which includes *alkB* (alkane-1-monooxygenase) and genes for beta oxidation, was inferred to be the most abundant amongst all hydrocarbon degradation pathways. *Klebsiella* sp. is the predominant ASV in all the sequenced consortia as well as the major contributor of hydrocarbon degradation genes. The findings of the study can serve as groundwork for the development of hydrocarbon-degrading bacterial consortia for the bioremediation of oil spill-affected areas in the Philippines. Likewise, this paper provides a basis for further investigation into the role of *Klebsiella* sp. in the bioremediation of hydrocarbon pollutants.

## 1. Background

Oil spills are one of the main culprits in the destruction of marine ecosystems, and their occurrence is relatively understudied in the Philippines. Despite this, the country is especially vulnerable to oil leaks because of the considerable maritime traffic it receives as an archipelago. Notably, the Iloilo Strait, which separates the islands of Guimaras and Panay, serves as a common route utilized by oil tankers transporting fuel between major population centers. On 11 August, 2006, the Guimaras oil spill accident occurred that was responsible for discharging roughly 350,000 tons of fuel oil approximately 200 km from the coastal areas of Guimaras Island and the neighboring Panay and Negros islands [1]. It contaminated about 450 hectares of protected mangrove forests, threatened Taklong Island National Marine Reserve [1, 2], and contaminated seafood products [3, 4], dubbing the incident as the most tragic oil spill in the Philippines.

In the aftermath of the Guimaras oil spill, traditional strategies such as spraying chemical dispersants and using spill booms were employed to prevent the oil from spreading further. In addition, siphoning of the oil was also done seven months after the oil spill. However, due to the vast area affected and rough sea currents, the oil decontaminating effort was unsuccessful [5]. Because of the inadequate efficiency and heightened costs of these strategies, bioremediation methods to remove oil spills should be explored. Bioremediation strategies for the removal of oil pollutants have proven to be efficient, cost-effective, and noninvasive compared to the physicochemical methods of detoxifying oil contaminants [6].

Autochthonous bioaugmentation utilizes native microorganisms in the polluted area for the removal of oil. This method for bioremediation is highly desirable as it minimizes the perturbation to the microbial ecology of the affected sites [7, 8]. Moreover, native microorganisms can better promote bioremediation efficiency as they tend to have a higher probability of surviving in the natural environment. Overall, microorganisms can utilize oil pollutants as a source of carbon and energy, which eventually leads to the mineralization of these contaminants into water, carbon dioxide, mineral salts, and biomass.

Oil spills result in the “bloom” of hydrocarbon-degrading species, which play a crucial role in the process of bioremediation. For instance, the genus *Oleispira*, an alkane degrader, was abundant at the site of the Deepwater Horizon blowout in the Gulf of Mexico [9]. Furthermore, alkane-degrading species from *Rhodobacteriaceae*, *Sphingomonadaceae*, *Chromatiales*, and *Actinobacteria*, and polycyclic aromatic hydrocarbon (PAH) degraders, such as *Pseudomonas stutzeri*, *Sphingomonas* sp., and *Tistrella mobilis*, were found to be abundant along the Spanish coastline affected by the oil spill from the Prestige oil tanker [10]. Due to the complexity of crude oil, bioremediation is best carried out by a community of microorganisms, with each member species responsible for the degradation of specific types of hydrocarbon depending on their respective enzymatic machinery [11]. Species involved in the bioremediation of oil from a polluted area can be enriched and then identified with the use of metataxonomics [12], which utilizes genetic markers, such as the 16S rRNA gene for bacteria and archaea, cross-referenced to a database. In addition, predictive analysis of the functional profile based on the amplified 16S rRNA sequences from the microbial community can aid in elucidating the role of the members of the consortium in hydrocarbon degradation.

Given that the Philippines is an archipelago, it is a hotspot for oil spill incidents that pose a substantial threat to its ecosystems. However, there is a paucity of information regarding the microorganisms suitable for bioremediation of oil spill-polluted sites in the country. Hence, the objective of this study was to enrich and characterize indigenous hydrocarbon-degrading consortia from oil spill-contaminated areas in Guimaras, an island province that is greatly impacted by oil spill incidents, for potential use in bioremediation.

## 2. Materials and Methods

### 2.1. Study Site Description and Sample Collection

**Figure 1:**
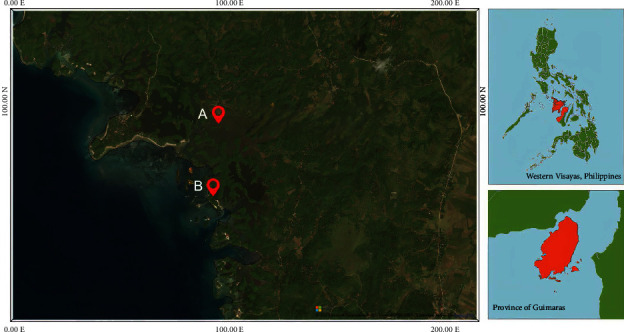
Location of the study sites in Brgy. Lucmayan, Nueva Valencia, Guimaras.

Soil samples were collected from two sites (Figure 1) in Brgy. Lucmayan, Municipality of Nueva Valencia in Guimaras. The GPS coordinates of A and B are 10° 27.8998′ and 122° 30.1661′ (51 N 445584.260 1156867.776 UTM) and 10° 27.6041′ and 122° 30.122′ (51 N 445502.961 1156322.974 UTM), respectively. The average soil temperature and pH during the time of sampling were 30°C ± 1°C and 7.1 ± 0.2 in Site A, while 30°C ± 1°C and 7.3 ± 0.1 in Site B.

Soil sampling was done at 12 : 00 pm PST (Philippine Standard Time), while it was still low tide. A total of 75 soil samples were gathered, 42 in A and 33 in B. Soil samples were collected using a small shovel to a depth of 10–30 cm. Ten grams of each soil sample were taken from each sampling point. The soil samples were placed in sterile Ziploc bags which were then put inside an ice chest.

### 2.2. Enrichment for Hydrocarbon-Degrading Consortia

The protocol for the enrichment of microbial consortia was carried out in Bushnell-Haas (BH) medium as previously described [13] with slight modifications. Soil samples were placed in a sterile 50 ml Falcon tube containing 30 ml of BH (composition in g/L: 0.2 g MgSO_4_; 0.02 g CaCl_2_; 1 g KH_2_PO_4_; 1 g K_2_HPO_4_; 1 g (NH_4_)_2_SO_4_; and 0.05 g FeCl_3_) medium. The BH was supplemented with 1.5% (v/v) diesel as the sole carbon source. The tubes were incubated at 30°C with shaking at 180 rpm for 7 days. After incubation, a 1% aliquot was transferred to the new BH medium with 1.5% (v/v) diesel and was incubated under the same conditions. The initial enrichment step was repeated for a total of three cycles (one cycle is equivalent to 7 days). After the initial enrichment, viable consortia were harvested through centrifugation (4,500 ×g, 15 min). The pellets were resuspended in fresh BH medium supplemented with 1.5% (v/v) diesel as the sole carbon source. These consortia were cryopreserved inside the −80°C biological freezer using 50% glycerol as the cryoprotectant.

### 2.3. The 2,6-Dichlorophenolindophenol (DCPIP) Assay

The 2,6-dichlorophenolindophenol (DCPIP) assay, as previously described in [14], was performed with minimal modifications to evaluate the ability of the enriched consortia in the degradation of hydrocarbons. The microbial consortia were precultured in 50 ml BH medium with 1.5% (v/v) diesel and were incubated at 30°C with shaking at 180 rpm until the optical density at 600 nm reached 1.0. Glucose as a carbon source was the positive control. The cultures were then centrifuged at 4000 ×g rpm for 5 min. The pellets were then resuspended in fresh BH medium, and the optical density (at 600 nm) was adjusted to 1.0. Afterwards, the following components were added in a microcentrifuge tube: 800 *μ*l of BH medium, 80 *μ*l of cell suspension, 4.5 *μ*l of 2,6-DCPIP solution (0.5% w/v), and 26.5 *μ*l of each hydrocarbon. The hydrocarbons tested were diesel, hexane, hexadecane, and xylene. The negative control set-ups were prepared without inoculum. The microcentrifuge tubes were incubated at 30°C for 7 days.

The 2,6-DCPIP is an artificial terminal electron acceptor that is blue in its oxidized state and colorless when it is reduced. A positive result for hydrocarbon degradation is indicated by a color change from blue to colorless, while a negative result would have no color change. To determine the percent degradation, each sample was centrifuged at 8,000 ×g for 15 minutes. The supernatant was analyzed at 609 nm using the UV-VIS spectrophotometer. The percentage of degradation values was computed using the formula [14] as follows:

(1) $$\begin{array}{c} \%\text{ degradation}=1-\left(\frac{A\text{bsorbance of treated sample}}{\text{Absorbance of control}}\right)x\;100\text{.} \end{array}$$

The computed percent degradations for diesel, hexane, hexadecane, and xylene were statistically analyzed using R [15]. The following model was used for the statistical analysis:

(2) $$\begin{array}{c} \left(\text{Percent degradation }∼\frac{\text{site}+\text{site}}{\text{consortia code}}\right)\text{.} \end{array}$$

The Shapiro–Wilk normality test was performed to assess the distribution of the data. A one-way analysis of variance (ANOVA) was conducted to assess for significant differences between the percent degradations of the consortia recovered from the two sites. Post hoc analysis via Tukey multiple comparisons of means was then accomplished.

### 2.4. Metataxonomic Profiling of the Hydrocarbon-Degrading Consortia

#### 2.4.1. Genomic DNA Extraction from the Hydrocarbon-Degrading Consortia

Total genomic DNA (gDNA) extraction from the consortium which obtained the highest percent degradation for each hydrocarbon tested was performed by Macrogen, Inc. (Seoul, Korea), following the protocol of the UltraClean® Microbial DNA Isolation Kit (MO Bio Laboratories, Inc., California, USA). The quantity and condition of the extracted gDNA were assessed using Victor 3 fluorometry and agarose gel electrophoresis, respectively.

#### 2.4.2. Amplification and Sequencing of V3-V4 Region of 16S rRNA Gene

Amplification of the V3-V4 region of the 16S rRNA gene and library preparation was also performed by Macrogen, Inc. (Seoul, Korea). The V3-V4 regions of the 16S rRNA gene of prokaryotes were amplified using primers 341F (5′CCTACGGGNGGCWGCAG-3′) and 805R (5′GACTACHVGGGTATCTAATCC-3′). Library preparation was carried out using Herculase II Fusion DNA Polymerase Nextera XT Index Kit V2, following the protocol of Illumina MiSeq 16S Metagenomic Sequencing Library Preparation Part #15044223 Rev. B.

#### 2.4.3. Microbial Community Analysis via QIIME2

The QIIME2 (Quantitative Insights into Microbial Ecology) pipeline was used for data preprocessing and taxonomic classification of the sequence reads from the samples. The sequence preprocessing step was done with the use of DADA2 denoise-paired pipeline to dereplicate sequences, remove chimeras, and to trim low-quality sequences. Chimera removal was done using the “consensus” setting of the denoise-paired pipeline. Subsequently, taxonomic assignment of representative sequences was done with the use of the SILVA 138−99 rRNA database using a Vsearch feature classifier (v2.7.0) using the default settings [16]. Uncategorized reads were then subsequently filtered from the sequences. Microbial diversity metrics such as alpha and beta diversity were then subsequently generated using QIIME2 software [17, 18]. The filtered outputs from DADA2 and the Vsearch classifier were exported to R using phyloseq, and compositional plots were generated using the Microbiome package [19, 20].

### 2.5. Predictive Functional Profiling of the Hydrocarbon-Degrading Consortia via PICRUSt2

PICRUSt2 (Phylogenetic Investigation of Communities by Reconstruction of Unobserved States) (version v2.0.3-b) was used for the prediction of the functional composition of the metagenomes [21]. The method used for the predictive functional profiling in environmental samples was adapted from the methodology that was previously described [22, 23]. The absolute abundance table of amplicon sequence variants (ASVs) generated from DADA2 was used as the input. Taxonomic classifications of each ASV were derived from the output from the Vsearch classifier. The “–stratified” option was added to the PICRUSt2 full pipeline to generate the individual contribution of each ASV for every predicted metabolic pathway. Postprocessing and visualization were carried out using R Studio [20].Processing was performed using dplyr and qiime2r, while visualization was accomplished with the use of ggplot2, heatmap.2, and microbiome [21, 22].

## 3. Results

### 3.1. Hydrocarbon Degradation by the Enriched Microbial Consortia

Thirty-two hydrocarbon-degrading consortia (16 each from sites A and B) were recovered from oil spill-affected sites after three cycles of enrichment cultivation on BH medium supplemented with 1.5% diesel, suggesting their ability to utilize diesel as a sole carbon source. The absence of growth on the other 43 consortia could be attributed to the limitations during the enrichment step (i.e., concentration of diesel, incubation conditions, salinity, and pH of the culture medium) or the inability to grow on the hydrocarbon.

**Figure 2:**
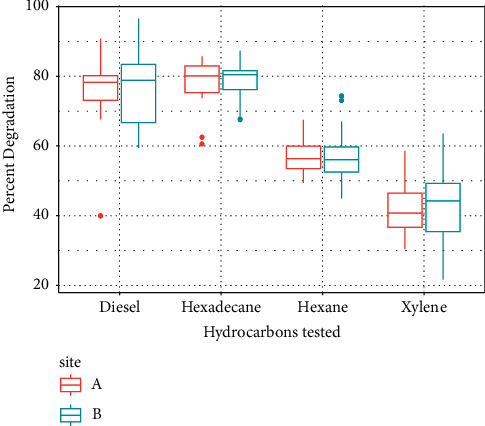
Box plot showing the percent biodegradation of the hydrocarbon substrates tested on the enriched hydrocarbon-degrading consortium via the 2,6-dichlorophenolindophenol (DCPIP) assay. The consortia were classified according to their site of origin. Bottom and top error bars indicate the 25th and 75th percentile per each degradation assay, respectively.

The 2,6-DCPIP assay revealed that all enriched consortia exhibited degradation activity on the four hydrocarbons tested. The consortium deemed as the best degrader for each of the hydrocarbons, and the respective percent degradation values were as follows: Consortium B2 for diesel (92% degraded), A5 for hexadecane (86% degraded), B1 for hexane (74% degraded), and B7 for xylene (63% degraded) (Figure 2). One-way ANOVA showed that between the two sites, only the percent degradations of the consortia during the diesel treatment had no significant differences (*P* = 0.745 > 0.5), while the rest of the hydrocarbon treatments produced a *p* value that is less than 0.5. Furthermore, post hoc analysis via Tukey's honest significant difference for hexane, hexadecane, and xylene revealed that consortia derived from site B had a higher overall percent degradation as compared to site A.

### 3.2. Metataxonomic Profiles of A5, B1, B2, and B7

**Figure 3:**
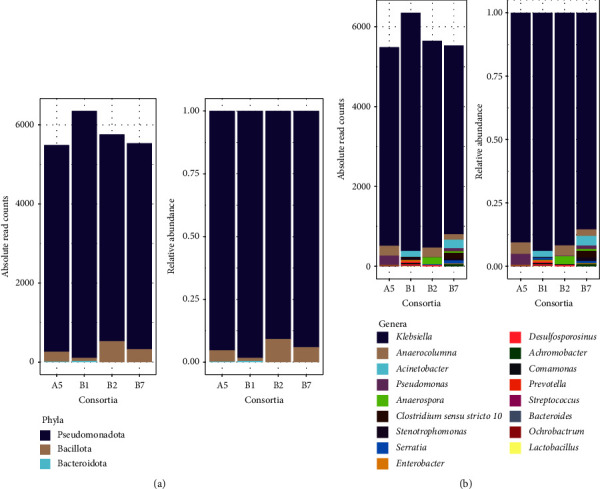
Metataxonomic profiles of A5, B1, B2, and B7. (a) Phyla. (b) Genera.

Metataxonomic profiles of the enriched consortia were composed predominantly of the phyla Pseudomonadota (between approximately 91% and 98%), followed by Bacillota (∼1% to 9%), and Bacteroidota (0.2% to 0.4%) (Figure 3). Classes Gammaproteobacteria and Alphaproteobacteria were represented under the phylum Pseudomonadota. Meanwhile, classes Clostridia, Negativicutes, Desulfitobacteriia, and Bacilli were present under the phylum Bacillota. The class Bacteroidia represents the phylum Bacteroidota. *Klebsiella* sp. (∼85% to 94%) is the most common and abundant genus in all four consortia. Other ASVs present in the enriched consortia were *Clostridium* sp., *Pseudomonas aeruginosa, Acinetobacter baumannii*, *Anaerospora* sp., *Clostridium sensu stricto 10, Acinetobacter calcoaceticus*, *Serratia* sp., *Stenotrophomonas* sp., *Enterobacter* sp., *Desulfosporosinus* sp., *Achromobacter* sp., *Acinetobacter* sp., *Comamonas aquatica*, *Prevotella* sp., *Stenotrophomonas maltophila*, *Streptococcus salivarius*, *Bacteroides* sp., *Ochrobactrum* sp., and *Lactobacillus* sp.

#### 3.2.1. Diversity Metrics of A5, B1, B2, and B7

**Figure 4:**
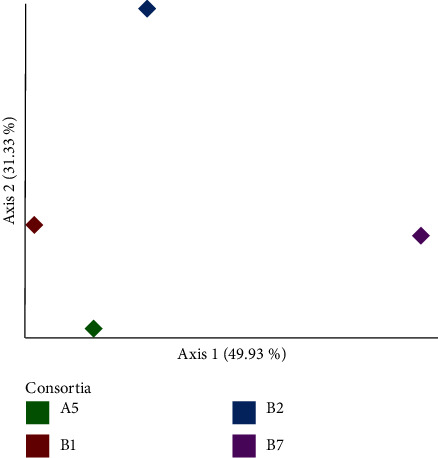
Principal coordinate analysis scatter plot showing the Bray–Curtis dissimilarity index of A5, B1, B2, and B7.

Consortium B7 contained the most varied species composition amongst all tested consortia, followed by B2 and B1 (Table 1). Consortium A5 had the least diverse species composition. Rarefaction curves revealed that all consortia were sufficiently sequenced and have captured their entire diversity. This was also supported by Good's coverage (*C*) values. This proves that all species detected in the four consortia make up 100% of the population. The Bray–Curtis dissimilarity index (Table 2 and Figure 4) suggests a high degree of similarity in the metataxonomic profiles amongst the consortia.

### 3.3. Predictive Functional Profiles of A5, B1, B2, and B7

**Figure 5:**
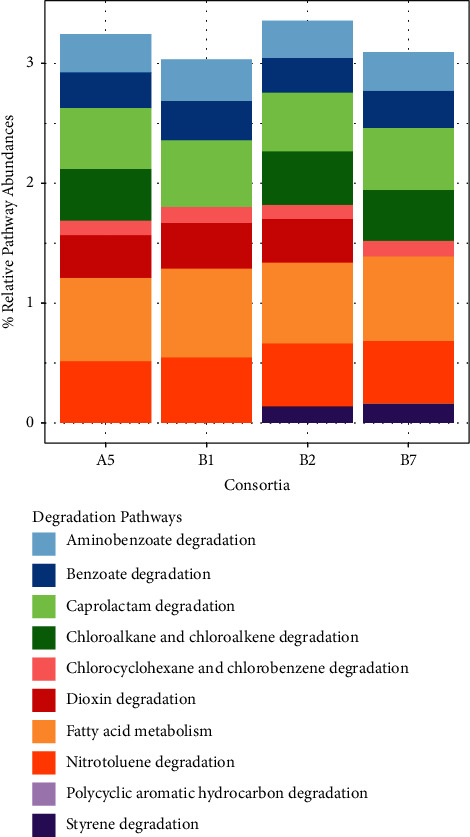
Predictive relative abundance of genes involved in the degradation pathway for xenobiotics present in A5, B1, B2, and B7.

**Figure 6:**
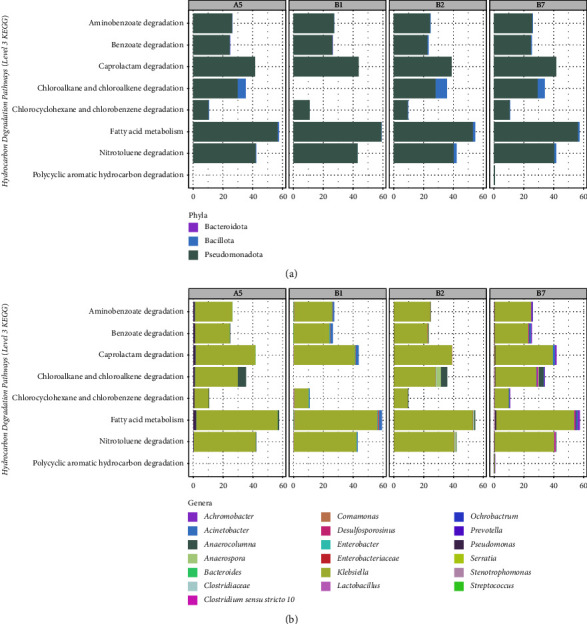
Prediction of the relative contribution of each ASV, binned according to phyla (a) and genus/family (b) for the hydrocarbon degradation pathways present in A5, B1, B2, and B7. The indicated counts shown in the plot were derived from the “Relative taxon abundance” column from the stratified PICRUSt2 output, which is the ASV's relative abundance in a given sample multiplied by the predicted function count found in the reference genome mapped to a given ASV. (a) Function relative abundance (per phylum). (b) Function relative abundance (per genus).

Predictive functional profiling revealed that all enriched consortia were equipped with genetic machinery to degrade xenobiotic compounds, including petroleum hydrocarbons. The mean weighted Nearest Sequenced Taxon Index (NSTI) of each of the 16S rRNA gene samples was 0.038 ± 0.007, indicating a high concordance between the reference sequences used by PICRUSt2 and the amplified 16S rRNA gene samples. A total of 190 KEGG orthologs (KOs) related to hydrocarbon degradation pathways were predicted to be present, namely, ko00627 aminobenzoate (30 KOs), ko00361 chlorocyclohexane and chlorobenzene (7 KOs), ko00362 benzoate (42 KOs), ko00625 chloroalkane and chloroalkane (14 KOs), ko00642 ethylbenzene (6 KOs), ko00061 fatty acid (23 KOs), ko00626 naphthalene (10 KOs), ko00633 nitrotoluene (18 KOs), ko00624 PAH (7 KOs), ko00624 toluene (13 KOs), and ko00622 xylene (12 KOs). The relative abundances of these pathways are shown in Figure 5. Amongst the 12 pathways for xenobiotic compounds degradation, eight are involved in hydrocarbon degradation. Notably, both pathways for aliphatic and aromatic hydrocarbons were predicted by PICRUSt2 in A5, B1, B2, and B7. Fatty acid metabolism, which includes *alkB* (alkane-1-monooxygenase) and genes for the beta oxidation pathway, was found to be the most dominant amongst all hydrocarbon degradation pathways. Other degradation pathways inferred were ascribed to benzoate, aminobenzoate, chlorocyclohexane, chlorobenzene, chloroalkene, chloroalkane, nitrotoluene, styrene, and caprolactam. PAH hydrocarbon degradation (% RPA = 0.008) was only present on B7, the best degrader for xylene. Furthermore, it was inferred that the phylum Pseudomonadota, specifically *Klebsiella* sp., mainly contributed to the potential ability to degrade different types of hydrocarbons (Figure 6).

## 4. Discussion

Microorganisms are key players in the biodegradation of hydrocarbon contaminants. The present study enriched hydrocarbon-degrading bacterial consortia from oil-polluted regions in Guimaras Island using BH medium supplemented with 1.5% diesel as the sole carbon source. The study demonstrated that all enriched consortia exhibit degradative activity to diesel, hexane, hexadecane, and xylene. The highest range of percent degradation (40% to 92%) was obtained when diesel was used as the sole carbon source, which was expected as the consortia were preconditioned in a diesel-containing medium during the enrichment process. Hexane, a short-chain n-alkane, was also degraded by the consortia with percent degradations ranging from 45% to 74%. Meanwhile, a larger overall percent degradation (61% to 87%) than hexane was obtained on hexadecane, a longer aliphatic hydrocarbon. Previous studies have argued that petroleum hydrocarbons with 5–10 carbon atoms are more detrimental to microorganisms since these compounds can destroy the organisms' lipid membranes [24]. Xylene, an aromatic hydrocarbon, was the most recalcitrant to biological degradation amongst the four tested hydrocarbons, with percent degradations ranging from 22% to 63% only. In general, aromatic hydrocarbons resist the biodegradation process and typically involve a multistep pathway that involves the addition of reactive side chains and the subsequent de-aromatization and ring cleavage [25].

16S rRNA gene sequencing revealed that Pseudomonadota species are well represented in the consortia, followed by Bacillota. Members of the phylum Pseudomonadota were proven to be pioneer oil degraders. A number of studies have revealed that Pseudomonadota is the most abundant phylum in environments contaminated with petroleum and in wastewater samples from petrochemical industries [26, 27]. Overall, ASVs retrieved were ascribed mainly to the Gammaproteobacteria class, whose dominance in enriched consortia is attributed to the fact that many hydrocarbonoclastic bacteria responsible for the first steps of hydrocarbon degradation are found in this class [28]. Species of the phylum Bacillota were highly ubiquitous and are considered as late stage-pioneer degraders of the more recalcitrant forms of hydrocarbons [29].

The findings of this study suggest a key role of *Klebsiella* sp. in hydrocarbon degradation based on its dominance in all the consortia. Consistent to this, *Klebsiella* sp. was previously reported to produce dioxygenases and hydroxylases that can break down alkanes [30]. Moreover, it can degrade a wide range of aliphatic and aromatic hydrocarbons and can produce biosurfactants [31]. Another study reported that oil field-adapted *K. pneumoniae* exhibited >90%, >70%, and >40% biodegradation rates for C10–C20, C21-C22, and C31-C32 hydrocarbons, respectively [32]. Notably, *Klebsiella* are known nitrogen-fixers, and studies have demonstrated that nitrogen-fixing hydrocarbon-degrading bacteria are significant members of consortia being used for oil bioremediation, since nitrogen is a common limiting compound in oil-contaminated sites [33].

Aside from *Klebsiella* sp., other ASVs of note include *P. aeruginosa* (A5, B7, and B2), *Acinetobacter* sp. (B1, and B7), *Serratia* sp. (B2 and B7), and *C. aquatica* (B1), which were previously reported in other studies as biosurfactant producers. Production of biosurfactants is advantageous in enhancing the biodegradation process as they increase the bioavailability of oil [34]. *P. aeruginosa* cells can use petroleum-based hydrocarbons as a carbon source. *A. baumannii* can degrade total petroleum hydrocarbon fractions of crude oil and is found to be a more vigorous biodegrader of crude oil than other biosurfactant-producing bacteria [35]. Similarly, another study [36] demonstrated that *A. calcoaceticus* can degrade aliphatic alkane hydrocarbons. Likewise, aerobic pathways for the removal of BTEX (benzene, toluene, ethylbenzene, and xylene) compounds are present in *Serratia* sp. [37]. *C. aquatica* was isolated previously from oil-polluted sites of Mumbai Harbor [38]. In addition, *Achromobacter* sp. (B2 and B7) and *Stenotrophomonas* sp. (B1, B2, and B7) are prolific PAH degraders. The former is a known degrader of polyaromatic hydrocarbons, particularly phenanthrene, naphthalene [39, 40], and chrysene [41], while the latter can degrade high molecular weight PAHs including phenanthrene [42], pyrene, benzopyrene, and benzoflouranthene [43]. 16S rRNA sequencing also identified strictly anaerobic species. The incubation conditions of BH broth, which became static at some point, must have influenced the presence of anaerobes. For instance, *Anaerospora* sp. (B2 and B7) is known to degrade chlorinated hydrocarbons, particularly aryl halides like chlorobenzene and chlorophenol. In one study, it was found to be the dominant member in the consortium isolated from sediments near an oil refinery at Yanbu Industrial City, KSA [44]. However, the presence of these abovementioned ASVs in the enriched consortia and their predicted degradation pathways contribution are seen to play a minor role only as compared to *Klebsiella* sp., whose relative abundances dominate across all sequenced consortia.

Species of bacteria possess distinct activities towards different types of hydrocarbon contaminants, which make a consortium more advantageous in the bioremediation efforts of petroleum hydrocarbons as opposed to monocultures. Consortia have a variety of catabolic genes, and the synergistic effects of these genes are favorable to attaining purification of hydrocarbon pollutants [33]. Furthermore, diversity metrics of each consortium as well as predictive functional profiling suggest that “generalist” microorganisms predominate across all sequenced samples. Generalist hydrocarbon degraders are species that can utilize other carbon sources to support their growth as opposed to obligate hydrocarbon-degrading bacteria (OHCBs) such as *Alkanivorax* sp. and *Cycloclasticus* sp. Because of the wider range of carbon sources that they can utilize, generalists tend to outcompete the OHCBs [45].

The number of ASVs detected in all four consortia in this paper was smaller compared to other enriched oil-degrading communities in other studies [13, 23], probably due to the higher final concentration of diesel used during enrichment. Enrichment processes result in a significant decline in the diversity index of the community, mainly due to a strong selection for hydrocarbon-degrading species. The species in the community are modified to favor the growth of those members that can survive in the modified environmental condition. In the same light, the toxic impacts of petroleum hydrocarbons could further reduce species richness, evenness, and phylogenetic diversity [46]. This is primarily linked to the disruption of the biogeochemical cycle of nitrogen because functional genes and species involved in nitrification are significantly lowered [23]. In addition, the limited oxygen concentration during incubation may have provided a selective advantage to certain microorganisms within the consortia. Thus, high-throughput screening methods may be utilized to enrich the functional resources of hydrocarbon-degrading bacteria. Meanwhile, the low dissimilarity indices amongst the taxonomic profiles of the consortia could be attributed to the similarity of the environments in which the consortia were derived from, as well as the uniform enrichment treatments that were carried out for all obtained samples.

Diverse genes for catabolism of aliphatic and aromatic hydrocarbons, especially different monooxygenases and dioxygenases, were predicted by PICRUSt2 in the consortia. These results corroborate the findings of another study [23] which by using PICRUSt2, predicted genes responsible for the degradation of aromatic hydrocarbons, including chlorobenzene, naphthalene, PAHs, ethylbenzene, benzoate, and xylene, in their enriched microbial consortia from similarly contaminated areas. By comparing the functional profiles of the natural to enriched microbial communities, the authors also found a significant increase in the hydrocarbon degradation pathways of enriched communities. This proves the importance of the enrichment process in generating a robust consortium equipped with the desired metabolic machinery. Moreover, complete degradation of hydrocarbon contaminants requires the collective efforts of different species in a microbial community. Overall, the PICRUSt2 pipeline suggests that bacterial communities in oil spill-affected areas adjust their metabolic pathways to hydrocarbon degradation.

The paucity of research on the prokaryotic communities and their functional diversity in oil spill-polluted areas in Guimaras makes it untenable to deduce the complete picture of the consortium dynamics from the time of the oil spill incident up to the present day. Assessing the microbial community profiles and their corresponding functional diversity is important in evaluating the recovery of oil spill-affected areas. For instance, the recuperation of the contaminated areas due to the Hebei Spirit oil spill incident in South Korea in December 2007 was determined by examining the community successions together with their respective functional profiles in the years 2014 and 2016. The findings for the aforementioned years demonstrated a higher abundance of microbial taxa associated with total petroleum hydrocarbon (TPH) degradation in 2014, but the taxa associated with PAH degradation were similar both years [47].

## 5. Conclusion

Bioremediation is a promising strategy to rehabilitate oil spill-affected areas. In this method, the metabolic potential of microorganisms is harnessed to degrade these toxic pollutants. The present study investigated consortia enriched from soil samples recovered from oil spill-affected sites in Guimaras, Philippines. The recovered consortia exhibit significant activity of degrading different classes of hydrocarbons, namely, diesel, hexane, hexadecane, and xylene, after multiple rounds of enrichment. Moreover, 16S rRNA gene sequencing showed that these consortia were composed primarily of generalist hydrocarbon degraders, with *Klebsiella* sp. dominating all characterized consortia. Predictive functional profiling using PICRUSt2 also inferred the presence of a number of relevant catabolic pathways for aliphatic and aromatic hydrocarbon degradation. *Klebsiella* sp. contributed mainly to the hydrocarbon-degrading capacity of each characterized consortium. This study, in turn, highlights the development of indigenous microbial consortia for the bioremediation of oil spill-affected areas. Likewise, this also warrants further investigation as to the specific functions of the member species in the consortia, especially *Klebsiella* sp., in the degradation of petroleum hydrocarbons. Lastly, additional research should incorporate *de novo* metagenomic approaches to study the potential functional profile of the enriched consortia from Guimaras, Philippines, with emphasis on their role in the degradation of petroleum hydrocarbons.

## Figures and Tables

**Table 1:** Alpha diversity metrics of A5, B1, B2, and B7.

Consortia	Chao1	Shannon	Inverse Simpson	Good's coverage
A5	19	3.942	0.762	1
B1	29	4.057	0.488	1
B2	32	4.178	0.469	1
B7	34	4.263	0.528	0.999

**Table 2:** Bray–Curtis dissimilarity index across A5, B1, B2, and B7.

	B7	A5	B1
A5	0.1408		
B1	0.1522	0.1026	
B2	0.1392	0.1258	0.116

## Data Availability

The 16S rRNA amplicon sequencing data generated in this study were deposited in the NCBI Sequence Read Archive (SRA) under accession numbers SRR23629379, SRR23629380, SRR23629381, and SRR23629382. The data are bundled under NCBI BioProject number PRJNA938983.
